# Validity and reliability of the CatWalk system as a static and dynamic gait analysis tool for the assessment of functional nerve recovery in small animal models

**DOI:** 10.1002/brb3.723

**Published:** 2017-05-18

**Authors:** Elisabeth A. Kappos, Patricia K. Sieber, Patricia E. Engels, Alessio V. Mariolo, Salvatore D'Arpa, Dirk J. Schaefer, Daniel F. Kalbermatten

**Affiliations:** ^1^ Division of Plastic, Reconstructive, Aesthetic and Hand Surgery Department of Surgery University Hospital of Basel Basel Switzerland; ^2^ Division of Neuropathology Institute of Pathology University Hospital of Basel Basel Switzerland; ^3^ Plastic and Reconstructive Surgery Department of Surgery, Oncology and Stomatology University of Palermo Palermo Italy; ^4^ Division of Plastic and Reconstructive Surgery Department of Surgery Ghent University Hospital Gent Belgium

**Keywords:** automated gait analysis system, dynamic and static gait parameters, peripheral nerve regeneration, rat and mouse sciatic nerve

## Abstract

**Introduction:**

A range of behavioral testing paradigms have been developed for the research of central and peripheral nerve injuries with the help of small animal models. Following any nerve repair strategy, improved functional outcome may be the most important evidence of axon regeneration. A novel automated gait analysis system, the CatWalk^™^, can measure dynamic as well as static gait patterns of small animals. Of most interest in detecting functional recovery are in particular dynamic gait parameters, coordination measures, and the intensity of the animals paw prints. This article is designed to lead to a more efficient choice of CatWalk parameters in future studies concerning the functional evaluation of nerve regeneration and simultaneously add to better interstudy comparability.

**Methods:**

The aims of the present paper are threefold: (1) to describe the functional method of CatWalk gait analysis, (2) to characterize different parameters acquired by CatWalk gait analysis, and to find the most frequently used parameters as well as (3) to compare their reliability and validity throughout the different studies.

**Results:**

In the reviewed articles, the most frequently used parameters were Swing Duration (30), Print Size (27), Stride Length (26), and Max Contact Area (24). Swing Duration was not only frequently used but was also the most reliable and valid parameter. Therefore, we hypothesize that Swing Duration constitutes an important parameter to be chosen for future studies, as it has the highest level of reliability and validity.

**Conclusion:**

In conclusion, CatWalk can be used as a complementary approach to other behavioral testing paradigms to assess clinically relevant behavioral benefits, with the main advantage that this system demonstrates both static and dynamic gait parameters at the same time. Due to limited reliability and validity of certain parameters, we recommend that only the most frequently assessed parameters should be used in the future.

## 1 INTRODUCTION

Over the years, different methods have been developed to analyze locomotor behavior in small laboratory animals like rodents. Measures of gait will be different at different velocities, such as before and after an injury, because walking slowly is another biomechanical entity than walking quickly. It is necessary to determine if the difference in gait measures results from the experimental setup, or rather from the animal choosing to walk at different speeds. New methods are able to fulfill this demanding task. Employing a treadmill, or limiting analysis to a narrow velocity window does address the effects of velocity (Kyriakou et al., 2016). Measuring across all velocities has been described though as more appropriate than dictating that the animals match speeds (Vandeputte et al., 2010). Quantifying locomotion with automated gait analysis devices is therefore a great way to evaluate the changes that experimental treatments provide, while simultaneously addressing the confound of many gait measures being velocity dependent (Neckel, 2015).

Nerve injuries usually lead to severe loss of a variety of functions. Due to the complex requirements for successful axonal regeneration, functional recovery is often poorly achieved. Experimental models are useful to investigate the mechanisms related to axonal regeneration and tissue reinnervation, and to test new therapeutic strategies to improve functional recovery. To assess regeneration and functional restitution after nerve injury, objective and reliable evaluation methods should be applied.

Navarro recently emphasized how important it is, to understand the main phases into which we can divide the process of nerve regeneration in order to select the most adequate methods and correctly interpret the results (Navarro, 2016). Those phases are regeneration of axons, reinnervation of targets, and recovery of functions. Each phase is dependent upon the preceding one for a successful outcome (Navarro, 2016).

The most relevant outcome of successful peripheral nerve regeneration is functional recovery. Recovery of function does not necessarily correlate with histological and electrophysiological evidence of regeneration (Munro, Szalai, Mackinnon, & Midha, 1998; Nichols et al., 2005; Valero‐Cabré & Navarro, 2002). Nevertheless, functional analysis offers the most reliable way to demonstrate that a nerve has not only regenerated, but also made useful end organ connections (Lee et al., 2009).

Objective evaluation methods should be used for the quantitative assessment of each of the phases of nerve recovery, and also for the investigation of the different types of functions (motor, sensory, autonomic; Navarro, 2016) It is advisable to use more than one functional method for each purpose, and also to perform morphological studies of the injured nerve and the reinnervated targets (Navarro, 2016).

Methods that focus on overground locomotion include contact electrode recordings, footprint analysis, 2D and 3D kinematics and over the last years the CatWalk (Afelt, Błaszczyk, & Dobrzecka, 1983; Chen , Tsai, Wang, et al., 2012a; Clarke, 1992; Clarke, Parker, & Smart, 1992; Górska, Zmysłowski, & Majczyński, 1999; Hamers, Lankhorst, van Laar, Veldhuis, & Gispen, 2001; Kunkel‐Bagden, Dai, & Bregman, 1993; Muir & Whishaw, 1999; Murrell et al., 1992; Thota, Watson, Knapp, Thompson, & Jung, 2005; Webb, Gowribai, & Muir, 2003).

The latter comprise a computer‐assisted gait analysis system that allows rapid and objective quantification of a large number of gait parameters, both static and dynamic. Especially dynamic parameters (e.g. swing duration, stance duration, and interlimb coordination) were difficult to analyze prior to the availability of CatWalk. The use of kinematics supports the analysis of dynamic parameters but is time consuming and the results are difficult to quantify. Consequently, CatWalk is one of the most suitable methods for quantitative analysis of overground locomotion in small animals like rodents.

### 1.1 History

The CatWalk gait analysis method and computer program was developed by Frank Hamers in 1996 in order to gain experience with the newly developed Basso, Beattie, and Bresnahan score (BBB score; Basso, Beattie, & Bresnahan, 1995). A complicating factor in scoring items in this test was the forelimb–hindlimb coordination (FL‐HL coordination), complicated by the fast movement of most animals so you can hardly ever see all four paws at the same time in the open field observation.

The original idea was to use a walkway to assess FL‐HL coordination. But it soon became apparent that the acquired runs on the walkway allow extensive and objective analysis of more gait parameters than just FL‐HL coordination. The wealth of gait‐related parameters that could be extracted from the runs through meticulous observation and the fact that the animals are crossing an elevated walkway like mannequins in a fashion show led to the name ‘CatWalk’.

In this study, we objectively quantified a large number of gait parameters during overground locomotion, using the CatWalk system in 42 articles published between 2006 and 2013 found, using the PubMed database. We qualified the most frequently used parameters and compared their reliability and validity throughout the different studies.

The aim of this article is to support a more efficient choice of parameters in future studies potentially adding to better interstudy comparability.

## 2 METHODS

### 2.1 Principle of operation

**Figure 1:**
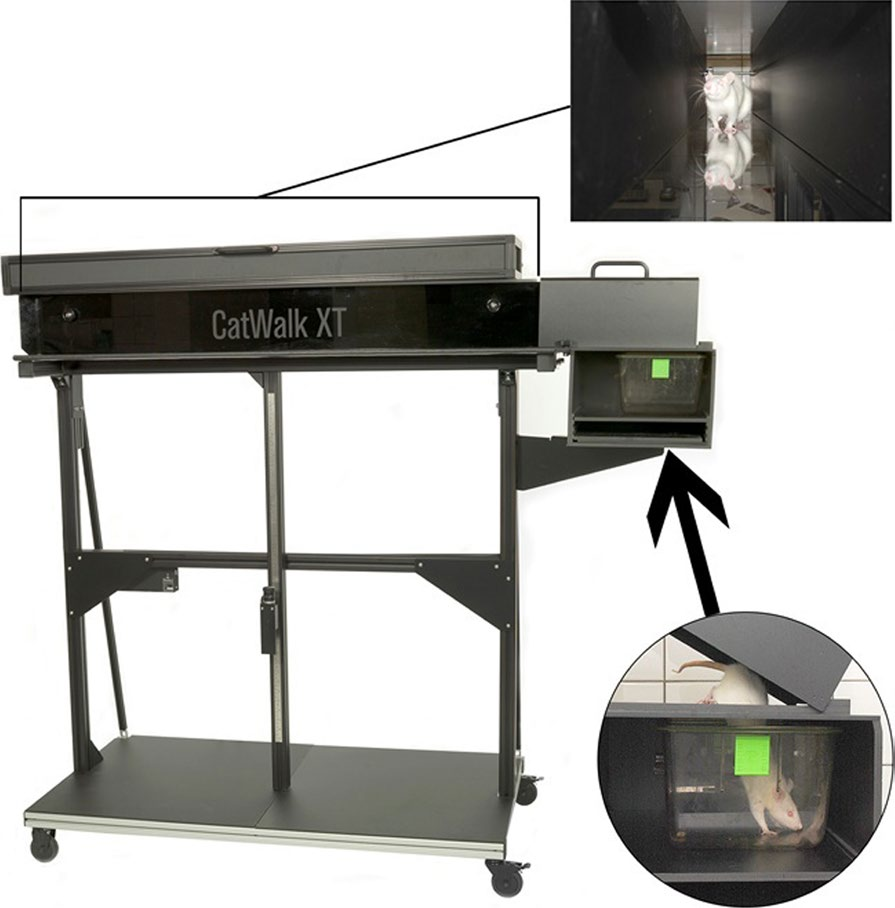
CatWalk Set Up: Animals cross the tunnel from left to right. Their footprints on the glassplate are detected by the camera below and transferred to the computer

The CatWalk would not be feasible without the walkway floor which consists of a piece of standardized glass with a thickness of at least 6 mm (Figure 1). The area where a paw contacts the floor is visualized, using an optical trick. An encased fluorescent tube is put alongside the distal long edge of the glass (as seen from the observer) so that light enters the glass from the edge (Clarke, 1992; Clarke et al., 1992). Some distance away from the edge, the light is completely internally reflected in the glass (same principle as fiber optics), except in those locations where air has been replaced by another medium such as a paw; there, light leaves the glass and illuminates the paw. In this way it is only the contact area that lights up clearly. Moreover, the applied pressure is representative for the intensity of the light, enabling the walkway measuring the force used by the paws (Hamers et al., 2001; Vrinten & Hamers, 2003).

Animals are filmed from underneath while crossing the walkway in a darkened room, thus providing good contrast between paw prints and the rest of the body. Fifty to sixty fields (half‐frames) per second are acquired, using a standard charge‐coupled device (CCD) camera, and are stored on a computer disk for off‐line analysis.

### 2.2 Parameters that can be assessed using CatWalk gait analysis

**Figure 2:**
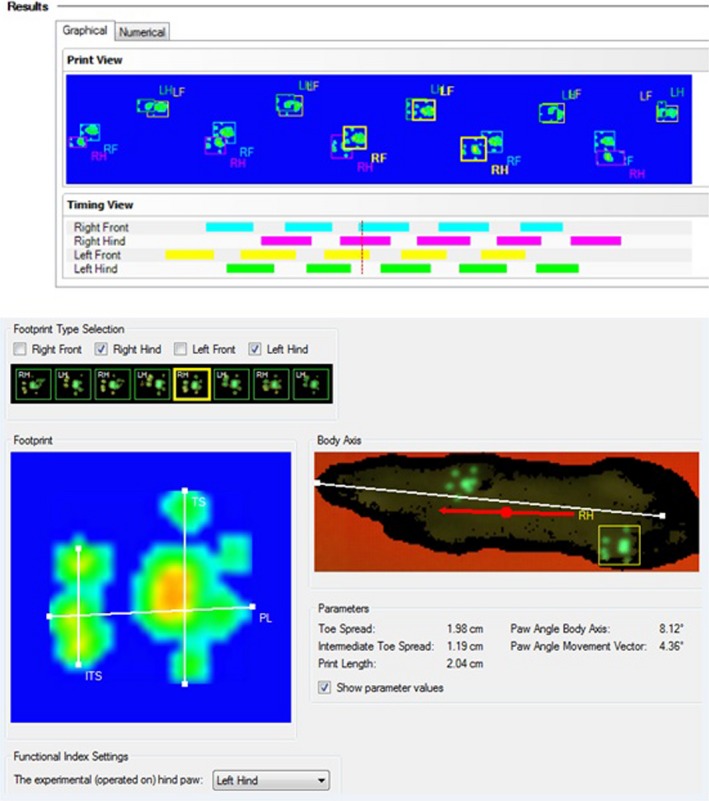
Data analysis by the CatWalk software: footprints are visualized on the screen, measurements taken and calculated to obtain different parameters

Data can be visualized and analyzed in different ways (Figure 2). First, it is possible to review the raw “videotape” of the walkway crossing in slow‐motion or frame by frame. As such, the program functions as a video recorder, albeit with the possibility to randomly assess individual frames. After the assignment of tags during the interactive analysis, stage gait can be qualitatively described by visualizing the walking pattern. This corresponds to the print pattern that would be left if the animal's paws had been dipped in four differently colored inks and the animal had run over a strip of paper. Prints can be inspected separately as well as in combinations, and timing diagrams for paw placements are available.

The parameters which can be assessed by using the CatWalk can be differentiated in “general parameters”, “qualitative data”, and “quantitative data”. In the following, we will focus on the last two.

#### 2.2.1 Qualitative data

Although not useful for statistical analysis, qualitative data such as replay of the walkway, crossing and print overviews supply insights into qualitative walking patterns (did the animal cross fluently, or did it hesitate, stop, rear?), and indicate the presence of gross gait abnormalities. The latter, if present, may suggest quantitative parameters that are of particular interest. It is possible to construct walking tracks such as those obtained by other methods, i.e., the de Medinaceli method, for any combination of paws, even fore‐ and hindpaws combined (de Medinaceli, Freed, & Wyatt, 1982). Printouts hereof might be used to compute the Sciatic Functional Index (SFI) and other parameters of interest in the sciatic nerve crush model, moving from qualitative to quantitative data.

#### 2.2.2 Quantitative data

Quantitative data is of most interest as it allows group comparisons and hypothesis testing. A large array of numeric outcome measurements can be obtained. Quantitative data can be divided into static paw parameters, dynamic paw parameters, coordination parameters, and locomotor speed.

##### Static and dynamic paw parameters

A large variety of measurements can be obtained from individual paws.

Among the most important are the *Print Size* and the width and length of the print, the *Stance and Swing duration* (and the hereof derived *Duty Cycle* and *Swing Velocity*), and two parameters measured during stance at the particular moment with the largest degree of paw ground contact: these are the *Max Contact Area* and the *Average Intensity*. The latter is especially important, as it depends on the pressure exerted by the paw during locomotion and can be used for assessing mechanical allodynia (Vrinten & Hamers, 2003). *Base‐of‐support, Stride Length* and *Relative Paw Position* are other parameters of interest.

Essentially, base‐of‐support measures the average width of the track made by the animal's forefeet and hind feet. The Stride Length simply expresses the length of a stride in millimeters.

The Relative Paw Position represents the relative positions of fore‐ and hind paws in which the hind paw position is related to the previous forepaw position. If the hind paw is placed after the forepaw, the distance counts as positive, else as negative. Most rodents tend to place their hind paw at the previous position of the forepaw, thereby maximizing the chance that the hind paws can be placed safely on the ground (e.g. without encountering a hole or a thorn). With pathology, this ability is often lost and a rather sensitive test in this respect is the horizontal ladder or grid walk test. The CatWalk makes it possible to quantify the distance error made by the animal.

##### Coordination parameters

The regaining of coordinated locomotion is an important milestone in recovery from nerve injuries. A precondition for coordination is the communication between different parts of the locomotor system. Therefore, investigation of coordination can yield important information about functioning of such connections. One of the most important scores for the coordination parameters is the BBB score.

In the BBB score, FL‐HL coordination is operationally defined as follows: for every FL step, a HL step is taken and HLs alternate. It is measured only when rats take three or four sequential steps. This particular definition was chosen as it can be assessed by experienced observers in the open field, without requiring specialized setups (Basso et al., 1995). Even then, open field FL‐HL coordination can only be assessed if the animal crosses path without hesitation and the frequency of such crossings decreases with time spent in the open field. Therefore, investigators tend to assess a limited number of crossings (often three or more) early during testing; if the three runs are coordinated, consistent coordination is considered (although strictly construed, at least 19 out of 20 coordinated runs are needed).

Apart from the definition used in the BBB scale, there are other ways of measuring coordination:

- Regular Step Pattern and Regularity Index

One other way of measuring coordination is the regularity index (RI), which relies heavily on the concept of regular step patterns. For fully coordinated locomotion, each paw is placed exactly once every four steps. There is a total of six possible step sequence patterns that can be used by a rodent while walking. These patterns can be categorized into 3 groups: alternate (Aa: [RF: right front‐RH: right hind‐LF: left front‐LH: left hind]: RF‐RH‐LF‐LH, Ab: LF‐RH‐RF‐LH); cruciate (Ca: RF‐LF‐RH‐LH, Cb: LF‐RF‐LH‐RH); and rotary (Ra: RF‐LF‐LH‐RH; Rb: LF‐RF‐RH‐LH). The Ab pattern is the most commonly observed. Of course, the definition of regular step patterns is based upon walking; during the (faster) trot, diagonal paw‐pairs are placed around the same time, and during a pace, ipsilateral paws are placed simultaneously. If two paws become visible at the same moment, the program assigns precedence to the paw that occludes the largest area at that moment and so classifies all stride cycles as if the animal was walking. The definition of coordination used for computation of the RI states that each completely coordinated walkway crossing consists of a succession of four to six regular step patterns without intervening missteps. The larger the number of missteps intersperse between regular step patterns, the lower is the RI. Although certainly useful in some respects, care must be taken in interpreting the exact value of this measurement. Points of concern are that animals can switch between different paw placement sequences and still be coordinated according to the normal walking pattern. To complicate things further, even uninjured animals occasionally make an error (Hendriks et al., 2006; Koopmans et al., 2005). Therefore, the control RI can be lower than 100%.

- Phase relationships as measures for coordination

A complementary way of describing interlimb coordination consists of using time relationships between footfalls. Hildebrandt, one of the pioneers of walking track analysis, already used such relationships to differentiate the gait of hundreds of animal species (Hildebrand, 1996). However, Hildebrandt failed to coin a short and easy name for this time relationship, which has led to some confusion. Gorska defined the diagonal phase shift as the ratio of the time interval between the onset of stance in a forelimb and the opposite hind limb to the duration of the forelimb stride cycle (Górska et al., 1999). Unfortunately, in later publications, the parameter was called “time shift” and in a recent study on treadmill locomotion in intact and spinal mice, this parameter was labelled as “coupling,” because it describes how movements of one leg are coupled to movements of the other leg (Bem, Górska, Majczyński, & Zmysłowski, 1995; Leblond, L'Esperance, Orsal, & Rossignol, 2003). To complicate things further, Kloos calls this parameter “phase dispersion”(Kloos, Fisher, Detloff, Hassenzahl, & Basso, 2005). The data recently published by Kloos was computed by hand from data gathered with the CatWalk program. There, the set of rules developed for these computations is now implemented under the heading “phase dispersions”. A much simpler computation of phase lags, but still incorporating the original notion of misses is also provided in recent publications under the heading “couplings” (Leblond et al., 2003).

##### Locomotor speed

Locomotor speed indicates the average walkway velocity and is calculated by dividing the distance of the walkway through the time the animal needed to cross. It describes a basic gait parameter that is generally neglected in most studies of movement disorders in neurological diseases and peripheral nerve studies. However, locomotor speed is known to affect several gait parameters in quadruples (Dellon & Dellon, 1991; Górska et al., 1999; Walker, Evans, Meade, Resig, & Sisken, 1994).

In the previous part, we described all parameters used in the reviewed publications. Each publication was also reviewed for evaluation of reliability and validity of CatWalk measurements compared to other outcomes measured (eg histological analysis). Furthermore, we identified the most frequently used parameters in the chosen articles and assessed them for reliability and validity. We judged validity as the applicability of the conclusion drawn from the measurements obtained by the CatWalk in each reviewed article. Finally, its a question if (and further to which degree) conclusions and data drawn from the specific experiment can be extrapolated outside of the lab. Reliability or reproducibility of the tested parameters was judged by yielding the same or compatible results in different experiments within the same study.

In addition, all manuscripts were analyzed for different nerve injury models to account for correlation between reliability/validity of CatWalk parameters across different injury models. The analysis was done by three of the authors independently.

## 3 RESULTS

All reviewed parameters are listed in Table 1. Table 2 contains all publications included in the parameter analysis of this review and further indicates which parameters were analyzed in each publication (Abada, Schreiber, & Ellenbroek, 2013; Baiguera et al., 2012; Balkaya, Kröber, Gertz, Peruzzaro, & Endres, 2013; Cao et al., 2008; Cendelín, Voller, & Vozeh, 2010; Chen, Tsai, Lin, et al., 2012b; Cheng et al., 1997; Cho, Nguyen, Satkunendrarajah, Branch, & Fehlings, 2012; Chuang et al., 2010; Côté, Detloff, Wade, Lemay, & Houlé, 2012; Deumens, Jaken, Marcus, & Joosten, 2007; Deumens et al., 2006, 2013; Encarnacion et al., 2011; Ferdinandusse et al., 2008; Gabriel, Marcus, Walenkamp, & Joosten, 2009; Gensel et al., 2006; Hausner et al., 2012; Hetze, Römer, Teufelhart, Meisel, & Engel, 2012; Hill, Brodak, & Bartlett Bunge, 2012; Hilton et al., 2013; Hoffmann et al., 2010; Jeong et al., 2011; Lee, McKeon, & Bellamkonda, 2010; Lin et al., 2010; Matsuura et al., 2013; Mountney, Leung, Pedersen, Shear, & Tortella, 2013; Neumann et al., 2009; Petrosyan et al., 2013; Schira et al., 2012; Sheu et al., 2012; Singh, Murray, & Houle, 2011; Sucher et al., 2010; Truin et al., 2009; Vandeputte et al., 2010; Vlamings et al., 2007; Wang et al., 2011, 2012; Westin, Janssen, Sager, & Temel, 2012; Yamamoto, Okui, Tatebe, Shinohara, & Hirata, 2011). Here 22 out of 42 analyzed papers showed high reliability and validity of at least one parameter measured by the catwalk. In 10 papers the reliability and validity was low, in the remaining 14 neither reliability nor validity could be identified. Swing duration (71%) and Stride length (62%) were the most frequently assessed parameters. Out of all the parameters, swing duration was the most reliable one with also the highest validity. Stride length and regularity index were the other two parameters with high reliability and validity. Parameters with high reliability showed catwalk results which correlated well with other accepted measurements. Simultaneously, the catwalk resulted in well‐expected outcomes, adding to the validity of its parameters. The 12 studies used peripheral nerve injury models (all of them sciatic nerve injury models): nine of which with high validity and reliability (75%), nine spinal cord injury models (four contusion injury and five traumatic transection injury models): four of which with high validity and reliability (44%), five degenerative disease models: two of which with high validity and reliability (40%), one hydrocephalus model (0%), three genetically modified models (0%), seven brain injury models (71%), and 1 arthritis model (0%).

**Table 1:** Parameters analyzed in the reviewed articles

General	
Body weight	Weight (g) of the animals
Dynamic paw parameters	
Stance duration	Duration (ms) of the stance phase
Swing duration	Duration (ms) of the swing phase
Step cycle duration	Duration (ms) of the step cycle (=stance + swing duration)
Static paw parameters	
Base‐of‐support	The distance (mm) perpendicular to the trajectory of movement between limb pairs, i.e. between the two forelimbs or between the two hind limbs.
Stride length	Length (mm) of a stride
Maximum Contact Area	The total floor area contacted by the paw during the stance phase; expressed in square pixel.
Relative paw position	Relative positions of fore‐ and hind paws: Hind paw position is related to the previous forepaw position. If the hind paw is placed (partially) after the forepaw, the distance is positive, else negative.
Coordination parameters	
Regular step patterns (RSP)	The definition of regular step patterns is based upon walking; during the (faster) trot, diagonal pairs are placed almost simultaneously. If two paws become visible at the same time, the program assigns precedence to the paw that occludes the largest area at that moment.
Regularity index (RI)	The RI defines coordination as the exclusive use of regular step patterns during uninterrupted locomotion. The RI grades the degree of coordination as follows: RI = [(RSP × 4)/PP] × 100%. PP represents the total number of paw placements (Górska, Bem, Majczyński, & Zmysłowski, 1993).
Phase lags	Another measure for interlimb coordination based upon time‐relationships between foot‐falls; the moment of initial contact of one paw is related to the stride cycle of another paw (Hendriks et al., 2006; Kloos et al., 2005; de Medinaceli et al., 1982).
Locomotor speed	The average speed of walkway crossing (cm/s) was calculated automatically by dividing the covered distance (cm) of the walkway through the time (s) needed to cross it.
Functional indices	
Sciatic functional index (SFI)	Index including print length on both the experimental and the normal sides, toe spread between the first and fifth digits on both sides, and the distance between the middle of the second and the fourth toes on both sides
Static sciatic index (SSI)	Index containing the ratios of hind foot parameters (1–5 toe spread factor [TSF] and intermediate toe spread factor [ITF]) of both injured and uninjured paws (Bervar, 2000).

**Table 2:** List of publications included in the analysis with parameters used

	Max. Contact Area	Print size	Stance duration	Swing duration	Step cycle duration	Base of support	Stride length	Relative paw position	Regular step pattern	Regularity Index	Phase lags	Locomotor speed	Rodents (Mice/Rats)	Reli‐ability	Validity	Sciatic functional index	Static sciatic index
30 Y. Matsuura: Peripheral Nerve Injury (PNI)	X	X	X	X									R	X	X		
31 H.A. Petrosyan: Spinal Cord Injury (SCI)						X	X						R	X	X		
32 B.J. Hilton: SCI	X	X		X		X	X					X	M	X	X		
33 J. Schira: SCI	X	X		X		X		X	X	X			R	X	X		
34 M.L. Sheu: PNI	X	X	X	X		X				X			R	X	X	X	
35 X.H. Wang: Degenerative Disease (DD)			X	X	X		X		X			X	M	X	X		
37 M.‐P. Côté: SCI			X	X				X		X			R				
38 R. Deumens: SCI	X	X				X				X			R				
41 Y.K. Abada: genetically modified (GM)	X	X	X	X		X	X	X		X		X	M				
42 A. Mountney: brain injury (BI)		X		X			X						R	X	X		
43 C. Baiguera: GM	X	X		X		X							M				
44 T. Hausner: PNI		X	X	X			X						R	X	X		
45 S. Hetze: BI	X	X		X	X	X	X			X			M	X	X		
46 N. Cho: SCI		X		X		X	X			X			M				
47 C.E. Hill: SNI							X	X		X			R				
48 S.H. Chen (b): PNI	X	X					X						R	X	X		
50 M. Balkaya: BI	X			X	X		X				X	X	M	X	X		
51 M. Yamamoto: PNI	X	X	X	X									M				
52 A. Singh: PNI				X		X	X			X			R	X	X		
53 A. Encarnacion: BI	X	X	X	X	X	X	X	X		X		X	R				
54 M. Jeong: CI				X			X			X			R	X	X		
35 Y. Wang: BI	X	X											M				
49 S.H. Chen (a): PNI	X	X					X						R	X	X		
55 J.E. Westin: DD			X	X	X	X	X	X		X		X	R	X	X		
56 C.‐S. Chuang: DD	X			X		X		X		X			R	X	X		
57 R. Sucher: PNI			X	X	X								M	X	X		
58 M. Hoffmann: Arthritis Induction (A)		X	X							X			R				
59 K.‐L. Lin: PNI	X	X	X	X		X			X	X			R	X	X	X	
2 C. Vandeputte: BI		X	X	X			X						R	X	X		
60 J. Cendelín: GM	X					X	X	X		X		X	M				
61 M. Truin: PNI	X		X	X									M				
62 H. Lee: SCI		X				X	X						R				
74 M.‐J. Lee: Hydrocephalus: H							X	X	X				M				
63 A.F. Gabriel:PNI		X	X	X	X								R				
75 A. Bozkurt: PNI	X	X	X	X									R	X	X	X	X
64 S. Ferdinandusse: DD	X	X	X			X							M				
65 M. Neumann: BI	X	X	X	X			X	X		X		X	M	X	X		
39 R. Deumens: PNI	X	X	X	X			X			X		X	R				
66 Y. Cao: DD				X		X	X		X	X		X	R				
67 R. Vlamings: DD	X		X	X		X	X		X	X		X	R				
40 R. Deumens: SCI		X	X	X		X	X					X	R				
68 J. C. Gensel: SCI	X	X					X		X			X	R	X	X		
Total	24	27	21	30	7	20	26	10	7	20	1	13	42	22	22	3	1

## 4 DISCUSSION

The recovery of sensory–motor functions after nerve injury requires that the nerve fibers regenerate and reinnervate adequate target organs in the periphery. A satisfactory functional recovery is not always achieved, despite effective regeneration and reinnervation. One of the reasons is that in complete transection of the peripheral nerve, axons grow at random and misalignment and inaccurate reinnervation are a rule. Thus, the degree of functional recovery depends on the type of injury and the type of repair, but also on how demanding a task is being used for testing (simple functions recover better than complex ones). Analysis of walking pattern via the recording of footprints is a well‐established and widely employed method for the assessment of motor recovery after nerve injury (Navarro, 2016), Translating the coordinate frame improves the ability to measure changes in base of support following nerve injury. Employing a treadmill, or limiting analysis to a narrow velocity window does address the effects of velocity (Kyriakou et al., 2016). But measuring across all velocities is more appropriate than dictating that the animals match speeds (Vandeputte et al., 2010). It is believed that quantifying locomotion with automated gait analysis devices like the CatWalk is a better way to evaluate the changes that experimental treatments provide and allows for a more appropriate way to address the confound of many gait measures being velocity dependent (Neckel, 2015).

A relevant point when studying novel strategies to promote nerve regeneration is that the methods selected allow serial evaluation of regeneration and reinnervation at desired intervals without having to execute the animals. Originally developed for analysis of locomotor function in spinal cord injury animal models, CatWalk has already proven its value not only in the functional evaluation of the peripheral nervous system (PNS), but also in different models of lesions of the central nervous system (CNS). The parameters collected through gait analysis are numerous and can be combined with each other to obtain the best analysis possible. Nevertheless, our paper shows that some of those parameters are used more frequently than others in functional nerve recovery models. Two reasons can be found:

First, some parameters, if combined, provide a good analysis of the level of dysfunction or, to the contrary, the level of functional recovery in the model studied.

Second, as described, gait analysis represents the result of different collaborating systems (CNS, PNS, muscles and joints) but is also highly dependent on the animal's behavior during examination (i.e. constant locomotor speed, trajectory). The latter is not easily managed which may explain why some of the CatWalk results are inaccurate (false positive/false negative). Therefore, it is favorable to choose parameters independent of such confounders to obtain more accurate results.

The possibility to test animals moving at different velocities and for longer time periods should greatly enhance the ability to differentiate between normal and pathological locomotion. But, as explained, in particular for phase lags, the results change significantly if the animal changes speed. That might certainly be one of the main reasons why this is beyond the less used parameter in the analyzed articles.

Despite the extensive use and literature on the walking track method, the results have been relatively disappointing (Varejão, Meek, Ferreira, Patrício, & Cabrita, 2001). First, the measurements of the footprints are, for different reasons, often difficult (Dellon & Mackinnon, 1989). Second, in contrast to the good return to near‐normal values after sciatic nerve crush, recovery is minimal and nondiscriminative between treatments after complete nerve section followed by any type of repair (Dijkstra, Meek, Robinson, & Gramsbergen, 2000; de Medinaceli et al., 1982; Shenaq, Shenaq, & Spira, 1989; Valero‐Cabré & Navarro, 2002). This is mostly attributable to the misdirection of regenerating axons and inaccuracy of muscle reinnervation after nerve section. The use of individual footprint measures rather than the complete SFI has been proposed to further increase the detection of changes (Hare et al., 1993; Munro et al., 1998). The Catwalk and similar systems with video recordings also enable the analysis of quantitative gait parameters as well as kinematic reconstructions of paw excursion. Despite the cost of their computerized systems, they have the advantage of being able to measure both dynamic and static gait parameters, assessing complex behavioral recovery of locomotion and allowing detection of small changes over time that are undetected by the earlier walking track methods.

As shown in the table, the most used parameters in the reviewed articles are Swing Duration (30), Print Size (27), Stride Length (26), and Max Contact Area (24). Swing Duration was not only frequently used but was also the most reliable and valid parameter.

It is important to underline their strength as demonstrated by the fact that they are frequently used simultaneously in different studies. The higher frequency of use of these parameters compared to other analyzed parameters is substantial and adds to a better interstudy comparability where those same parameters were used.

The frequently used parameters are conducive to study models of CNS diseases like Parkinson disease, Huntington Chorea and, moreover in models of cord injuries and peripheral nerve regeneration, but can also be assessed for other experimental disease models, like arthritis or muscular disorders.

Only 22 out of 42 reviewed studies (52%) showed a high reliability and validity. This suggests that not only functional analysis but a combination of different outcome measurements are crucial for interpretation of experimental data. For optimal data analysis, the Cat Walk should be combined with other functional tests. Comparison of the time and completeness of regeneration, reinnervation, and functional recovery by different types of peripheral nerve fibers is possible by using several neurophysiological methods. Variable results between different methods applied to evaluate nerve regeneration in the same animals are not unexpected. The variability may be due to the type of fibers assessed, the complexity of the functional response, and the ability of the methods to detect the response.

The horizontal ladder inclined rolling ladder and the Catwalk together may prove useful for multimodal functional recovery testing after many experimental nerve injuries. It is generally recommended to use more than one functional method for each purpose, and also to perform morphological studies of the injured nerve and the reinnervated targets (Navarro, 2016).

## 5 CONCLUSION

For a multimodal analysis approach, it is in general recommended to combine electrophysiological and behavioral tests that can be repeatedly performed along follow‐up, with histological study of the regenerated nerve and immunohistochemical studies of target reinnervation. The results of each test may not necessarily correlate with those of other tests, particularly after severe nerve lesions, but together allow for a comprehensive quantitative evaluation of the complex process of nerve regeneration (Bozkurt et al., 2008; Kappos et al., 2015; Navarro, 2016).

Due to limited reliability and validity of certain parameters, we recommend that only the most frequently assessed parameters should be used in the future. Especially swing duration, but also stride length and regularity index are the most useful parameters. Even if CatWalk represents a valuable tool for gait analysis, major improvements in both the walkway setup and the ease of use of data categorization may be expected.

Our results confirm that choice of injury model greatly impacts alteration of CatWalk parameters.

We conclude that CatWalk can be used as a complementary approach to other behavioral testing paradigms to assess clinically relevant behavioral benefits, with the main advantage that CatWalk demonstrates both static and dynamic gait parameters at the same time (Bozkurt et al., 2008).

## CONFLICT OF INTEREST

None of the authors have any competing interests. We have no disclosures and none of the authors received any sponsoring by the company producing the CatWalk.

## AUTHOR'S CONTRIBUTION

EK, PS, PE, and AM carried out the literature research, participated in the analysis of the reviewed papers and drafted the manuscript. DK and DS participated in the design of the study. All authors read and approved the final manuscript.
